# Mechanosensitive Cation Channel Piezo1 Is Involved in Renal Fibrosis Induction

**DOI:** 10.3390/ijms25031718

**Published:** 2024-01-31

**Authors:** Marta Drobnik, Jakub Smólski, Łukasz Grądalski, Szymon Niemirka, Ewelina Młynarska, Jacek Rysz, Beata Franczyk

**Affiliations:** 1 Department of Nephrocardiology, Medical University of Lodz, ul. Żeromskiego 113, 90-549 Lodz, Poland; marta.drobnik@stud.umed.lodz.pl (M.D.);; 2 Department of Nephrology, Hypertension and Family Medicine, Medical University of Lodz, ul. Żeromskiego 113, 90-549 Lodz, Poland

**Keywords:** renal fibrosis, collagen, Yoda1 and Jedi1/2, GsMTx4, Piezo1 channel, ion channels

## Abstract

Renal fibrosis, the result of different pathological processes, impairs kidney function and architecture, and usually leads to renal failure development. Piezo1 is a mechanosensitive cation channel highly expressed in kidneys. Activation of Piezo1 by mechanical stimuli increases cations influx into the cell with slight preference of calcium ions. Two different models of Piezo1 activation are considered: force through lipid and force through filament. Expression of Piezo1 on mRNA and protein levels was confirmed within the kidney. Their capacity is increased in the fibrotic kidney. The pharmacological tools for Piezo1 research comprise selective activators of the channels (Yoda1 and Jedi1/2) as well as non-selective inhibitors (spider peptide toxin) GsMTx4. Piezo1 is hypothesized to be the upstream element responsible for the activation of integrin. This pathway (calcium/calpain2/integrin beta1) is suggested to participate in profibrotic response induced by mechanical stimuli. Administration of the Piezo1 unspecific inhibitor or activators to unilateral ureter obstruction (UUO) mice or animals with folic acid-induced fibrosis modulates extracellular matrix deposition and influences kidney function. All in all, according to the recent data Piezo1 plays an important role in kidney fibrosis development. This channel has been selected as the target for pharmacotherapy of renal fibrosis.

## 1. Introduction

From 10% to 14% of the global population is affected with chronic kidney disease with subsequent deposition of collagen that is considered as its hallmark of this process [1]. Fibrosis, known as the degenerative phenomenon of the connective tissue, comprises excessive accumulation of extracellular matrix (ECM) within the organ. Moreover, the crosslinking of collagen embraced in fibrotic changes is incorrect. A twofold meaning of fibrosis should be taken into consideration. Hence, this process is the result of tissue injury that initially induces a healing-type response aimed at fibrotic scar development. Fibrotic scar restores organ continuity or initiates its damage [2]. The augmentation of ECM accumulation within the kidney is the result of their injury followed by an inflammatory reaction. The inflammatory mediators stimulate the fibroblasts or myofibroblasts’ function that comprises collagen synthesis. Continuous deposition of ECM within the kidney restricts step-by-step renal function and leads to kidney failure.

**Figure 1:**
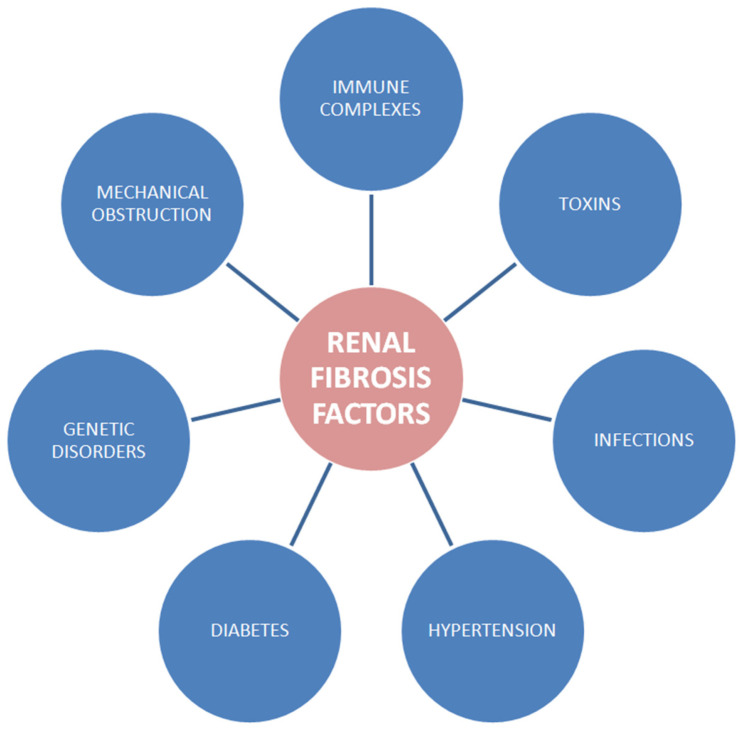
Factors of kidney fibrosis [4].

The three types of fibrotic deposits within the kidney were observed in the glomerulus, tubules and vasculature [3]. Several pathological stimuli such as infections, toxins, mechanical obstruction, immune complexes as well as genetic disorders lead to injury of cells and they may induce fibrogenesis (Figure 1). Additionally, type-2 diabetes and hypertensive nephropathy may be linked with fibrosis of the kidney [4]. The fibrotic process is associated with leukocyte recruitment, angiogenesis as well as myofibroblasts accumulation within the kidney interstitium. After kidney injury, macrophages, dendritic cells and T lymphocytes flow into the interstitium of the organ [5]. The influx of macrophages to the kidney accelerates a fibrotic process [6]. Moreover, the depletion of these cells’ number reduces the ECM accumulation within the kidney. Macrophages could be transformed by interferon-gamma (IFN-γ) (Table 1) and lipopolysaccharides into the M1 phenotype or by interleukins (IL) IL-4 and IL-13 into the M2 phenotype. The shift of macrophages from M1 to M2 is observed when the inflammatory step is moved into the phase of restoration of wound healing [4]. Furthermore, macrophages release the profibrotic cytokines [7]. Interstitial fibrosis impairs tubular function and leads to organ damage. Administration of diphtheria toxin (causing tubulo-specific injury) leads to proximal tube injury and induces inflammation, tubulointerstitial fibrosis [8] and even glomerulosclerosis.

After injury, the myofibroblasts appear within the interstitium of the kidney. These cells are able to synthetize collagen as well as contain within cytoplasm contractile microfilament bundles composed of α-smooth muscle actin and include additional markers such as vimentin and desmin [14]. The precursors of myofibroblasts within the kidney are a matter of debate. Thus, stromal cells, endothelial cells, leukocytes or epitheliocytes are hypothesized to be the source of myofibroblasts within fibrotic kidney [5].

Collagen accumulation is dependent not only on its synthesis but also on this protein breakdown. The cleavage of collagen is dependent on matrix metalloproteinases regulated by tissue inhibitors of matrix metalloproteinases. Furthermore, adamalysins, catepsins, meprins and plasmin participate in collagen breakdown [15].

The cellular function within the organ is regulated not only by biochemical cues but also by mechanical stimuli. Thus, the physical stimuli are recorded by the cells and then translated into the biochemical signals. The two types of mechanosensitivity were reported: the first comprises forces directly stimulating cells (tension, compression). The second type of mechanosensitivity is referred to the elasticity of a cell environment [16]. The elasticity of the environment is the ability of the material to deform under the influence of the force and the recovery of the material to the original shape when the force disappears. For quantification of the elastic properties of materials, Young’s modulus is used [17]. Mechanosensing comprises an assessment of the mechanics of the tissue via mechanoreceptors: cadherins, integrins, glycocalyx, tyrosine kinase receptors, G-protein coupled receptors, diskoid domain receptors—DDR as well as ion channels (Piezo, transient receptor potential ion channels, epithelial sodium channel superfamily). Mechanical environment influences cell phenotype. Changes in the tissue stiffness could be dependent on pathological processes leading to disease development [16,17].

Correct nephron function is also dependent on mechanical stimulation. Processes such as tubular flow, the pressure of fluid in kidney tubules or wall tension are detected by epithelial cells within the kidney [18]. In physiological conditions, Young’s modulus of kidney parenchyma is 4 kPa but during fibrosis, the tissue is replaced by a stiff extracellular matrix. The fibrotic kidney stiffness may rise up to 35 kPa [19,20].

## 2. Piezo1 Channel

**Figure 2:**
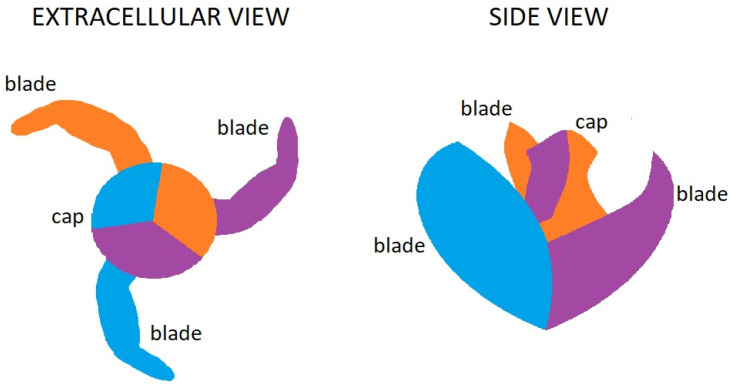
Scheme of structure of Piezo1 channel [9].

Piezo1 is the mechanosensitive nonselective ion channel, that plays an important role in several physiological processes. This protein is composed of over 2000 amino acids [21,22]. A central pore of the channel is covered by a single cap and trimers around it. The trimers are shaped like the blades of a propeller (Figure 2). This structure is fundamental for mechanosensing and support transduction [23,24]. Piezo1 is activated by the changes in membrane tension by physical forces such as shear stress, stretch, compression and osmotic stress. Activated Piezo1 channel is permeable to Na^+^, K^+^, Ca^2+^ and Mg^2+^ with a slight preference for calcium ions. Piezo1 is necessary for transduction of internally and externally applied pressure across the cell membrane [25]. Mechanical stimuli are converted by Piezo channels into biochemical information that is understandable for the cells.

Fu and coworkers imply that mechanical stimulus activates directly the channel without stimulation of other proteins or intracellular messengers [26]. Thus, Piezo channels are believed to be direct sensors of mechanical stimuli. On the other hand, the reception of physical stimuli could be also dependent on the conterminal components of the cellular membrane [27]. Two different models of Piezo1 activation are considered: force through lipid and force through filament. The force-through-lipid model suggests that Piezo1 gating depends on the changes in tension of adjacent lipid components of the cellular membrane [27,28]. This hypothesis is supported by an experiment showing the channel activation in the model of membrane bulbs that do not contain cytoskeleton [29]. The force-through-filament model highlights the connection of Piezo1 with the cytoskeleton. Piezo1 is localized close to the E-cadherin/β-catenin/F-actin complex and it is tethered to the actin cytoskeleton of the cells. Experimental impairment of cadherin-β-catenin mechanotransduction complex decreases a Piezo-dependent response. The extracellular domain of E-cadherin is attached to the Piezo1 cap domain which is responsible for controlling the transmembrane gate. Moreover, the cytosolic tail of E-cadherin interacts with the domain of Piezo1 localized in the cytoplasm. The cytosolic domain of Piezo1 is proximally situated to its intracellular gates. Interaction of the mentioned domains can support the gating of Piezo1. Augmented tension of the membrane depends on endogenous acto-myosin-dependent traction forces and this phenomenon can cause channel opening in situations when external stimuli are absent [30]. Moreover, the local tension applied to the membrane could be transmitted across the cytoskeleton and activate distant Piezo1 channels. This observation suggests that sensing of external matrix stiffness could be dependent on Piezo1 [31].

Piezo1 was reported to be highly expressed in the endothelial cells, kidney and bladder [26]. The channels influence vascular structure [21], blood pressure homeostasis [32], urine osmoregulation [33], bone remodeling and osteogenesis [34]. They also have an impact on the development of heart and kidney diseases [35]. Piezo1 protein appears within cortical and medullary collecting duct, proximal and distal convoluted tubule and the renal corpuscle [33,36]. Higher expression of Piezo1 was shown in the human renal tubule and the main collecting duct in fibrotic kidneys [9]. Piezo1 influences an expression of extracellular matrix such as collagen type II and IX within the kidney [26]. Mechanical stimuli activating Piezo1 channels exerted profibrotic effects in the human kidney 2 (HK2) and mouse proximal tubular cells (mPTC cells) [9].

## 3. Piezo2 Channels

The family of piezoelectric channels, including Piezo1 and Piezo2, plays a crucial role as mechanically sensitive transduction molecules in mammals. These channels are essential for converting mechanical signals into biological cues, thereby regulating numerous physiological processes [37].

Piezo2 has recently been identified as a mechanically activated ion channel, predominantly present in sensory neurons and other tissues. It exhibits specific expression in mesangial cells and renin-producing juxtaglomerular cells. Moreover, Piezo2 is found in neural structures associated with sensory reception, such as dorsal root ganglia, sensory nerve endings and Merkel cells [38]. Piezo2 plays a pivotal role as a sensory transduction channel, translating mechanical force acting on the cell membrane into ion influx. This, in turn, initiates a bioelectric cascade that translates mechanical stimuli into biochemical responses [39].

## 4. Pharmacological Inhibitors and Stimulators of Piezo Channels

Piezo were classified as the novel class of ion channels participating in mechanotransduction. Moreover, they participate in the regulation of some physiological processes not only within the kidney but in all organs. They can also be involved in the development of some pathological phenomena such as fibrosis, anemia [40] and neoplasms [41]. Thus, Piezo channels are considered to be a target for various disease therapies [40,41]. Pharmacological tools comprising both Piezo channel inhibitors as well as stimulators have been developed. They support significantly the experiments investigating the Piezo1 channel’s physiological role as well as its involvement in pathological processes. Among inhibitors, the following compounds are known: streptomycin and spider peptide toxin (GsMTx4). However, these inhibitors are not selective for the Piezo1 channel. Other ion channels are also blocked by these compounds. Nonspecific blockade of Piezo1/2 channels could be achieved by ruthenium red (RR) the polycation blocking only intward ion current when it is applied extracellularly. Contrary to that outward current and Piezo mediation, mechanically activated currents are not influenced by this compound [42]. RR was found to interact with two residues: E2495 and E2496 on Piezo1 and E2769, as well as E2770 on Piezo2 [43]. GsMtx-4 amphipathic peptide toxin is known as the inhibitor of Piezo channels. The inhibition of Piezo1 is reversible. Modulation of the cell membrane tension is supposed to be responsible for the action of GsMtx-4 [44]. Moreover, shear-stress-induced stimulation Piezo1 is blocked by enantiomeric amphipathic peptides AB [45]. Discovery of the specific antagonists will be the breakthrough in Piezo channels research. In spite of that fact, the currently available inhibitors are considered in the prevention of kidney fibrosis [46].

Piezo1 channels could be chemically activated by synthetic compounds such as Yoda1 or Jedi1/2 [46,47]. Yoda1 is the hydrophobic synthetic small molecule (compound 2-[5-[[(2,6-Dichlorophenyl)methyl]thio]-1,3,4-thiadiazol-2-yl]-pyrazine [11]), that mimics mechanical stimulation of Piezo1 channels as a selective activator. The effect of Yoda1 is not dependent on other membrane components and proteins. This compound is used to reveal the Piezo1 presence within cells and tissues [11,23]. Yoda1 has no impact on Piezo2 channels [48]. It is able to bind directly to proteins of mPiezo1. Doku1 (analog of Yoda1) antagonizes Yoda1 effect, but this compound itself does not have any stimulatory activity [49].

In 2018, new chemical activators of the Piezo1 channel were discovered. They were called Jedi1 and Jedi2 [46]. Both Jedi1 and 2 are selective activators of Piezo1. The two compounds are not effective in activation of Piezo2. The Yoda and Jedi act by different mechanisms [50]. These small molecules weigh 202.81 Da (Jedi1) and 208.23 Da (Jedi2). Their activating effect on Piezo1 is probably dependent on the 3-carboxylic acid methyl furan structural motif that is present in the two mentioned molecules. The lack of structural similarity between Jedi1/2 and Yoda1 suggests a different mechanism of Piezo1 activation. Jedi1/2 have better water solubility (up to ~2 mM) than Yoda1 (up to ~30 μM) [47]. The dissimilar response of the Piezo1 channel to Jedi and Yoda1 was observed. A prompt activation, apparent decay and rapid reversibility are typical for Jedi-induced response. Contrary to that, Yoda1-induced response comprises slow activation, slow reversibility and decay was not observed [46].

## 5. Piezo1 Role in Kidney Fibrosis

Several studies have shown that the Piezo1 protein is present in many types of tissues, for example in the kidney, and can be excited by mechanical stimuli [26]. Yoda1 (Table 1) is the most powerful Piezo1 activator acting in the mechanism of energetic modulation of mechanosensory domains [51]. The involvement of Piezo1 in the process of renal fibrosis was confirmed by experiments with the application of Yoda1. After activation of Piezo1, fibronectin, transforming growth factor β1 (TGF-β1) and collagen accumulation were elevated [9]. This effect was reversed by the application of GsMTx4 [9]. TGF-β1 (Table 1) is a key cytokine supporting the development of renal fibrosis [52]. TGF-β1 contributes to the elevation of ECM accumulation through increased synthesis of proteins such as collagen and inhibition of their degradation [9]. Furthermore, this cytokine induces tubular epithelial–mesenchymal transition [53]. Klotho-derived peptide 1 (KP1- an anti-aging transmembrane protein) is known as a coreceptor for Fibroblasts Growth Factor 23 and an inhibitor of TGF-β1 signal transmission to prevent kidney fibrosis. KP-1 blocks the attachment of TGF-β1 to its type 2 receptor (TβR2). A low level of KP-1 was reported in the fibrotic kidney. On the other hand, KP-1 preserves the function of the kidney and ameliorates kidney fibrosis after ischemia–reperfusion injury [54]. Other experiments performed on HK2 cells treated by Yoda1 showed an influx of calcium ions to the cells as well as augmentation of the fibrotic process. This study proved the involvement of Piezo1 in the induction of profibrotic changes in renal proximal tubular cells. The described above effect was dependent on the activation of the calcium/calpain2/integrin beta1 pathway [9].

### The Calcium-Calpain2-Integrinβ1-Fibronectin Pathway 

**Figure 3:**
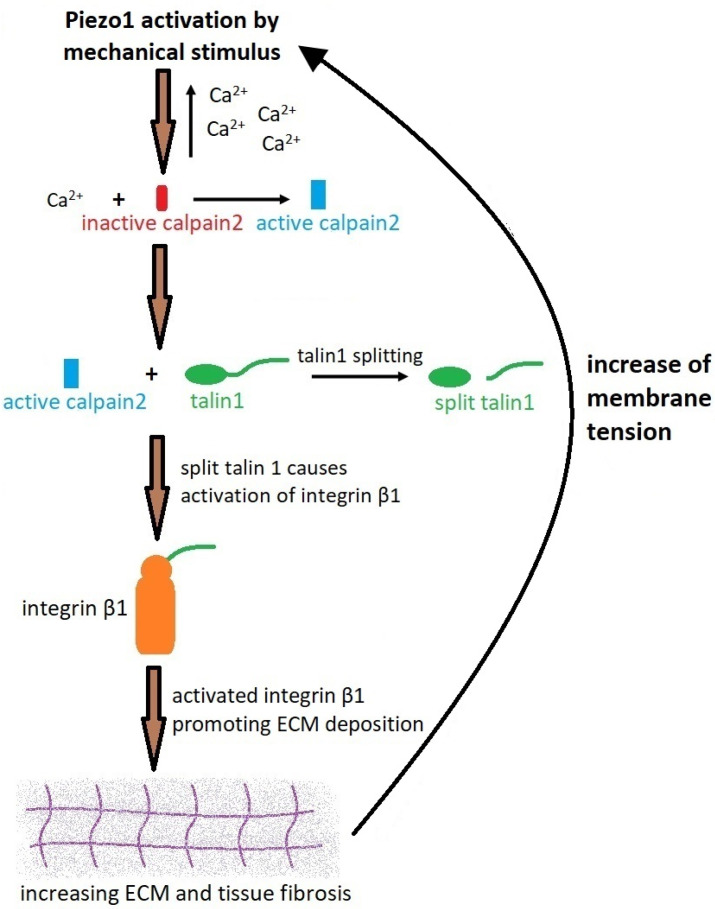
Piezo1 activation and the process of fibrosis. When the kidney becomes fibrotic, mechanical forces increase the tension of the tubular epithelial membrane, which is caused by the increased amount of ECM in the tubulointerstitial areas and increased pressure in the kidney. These changes lead to increased intracellular calcium influx through the Piezo1 activation. Ca^2+^ stimulates calpain2 which activates talin1. Active talin1 activates β1 integrin, which supports ECM development in the tissue. As a result of the activation of the Piezo1 protein, the synthesis of the TGF-β1 factor is increased, thereby enhancing the profibrotic effect. Consequently, increased amounts of ECM and fibrin cause an enhanced response of the Piezo1 protein to mechanical stimuli, leading to 10 further progressions of kidney fibrosis [9].

Activation of Piezo1 results in increased intracellular calcium influx in HK2 cells which consequently activates calpain2 (calcium-dependent protease) (Table 1) and initiates the profibrotic response (Figure 3). The calpain2 reaction, leading to calcium influx is mediated by the Piezo1 channel. This effect depends mainly on the extracellular concentration of these ions. Extenuation of the fibrotic effect following Yoda1 application was observed after pretreatment of the HK2 cell cultures by EGTA chelator of the calcium ions. Moreover, further studies performed on calpain2 knockdown HK2 cells showed a significant decrease in fibronectin and α-SMA levels after Yoda1 treatment. Observed effects support the hypothesis that calpain2 is an important target of Piezo1 [9,55,56]. Moreover, Yoda1 application to the cultures increased the amount of talin1, the constituent of the adhesion complex linking the integrin receptor with the cytoskeleton. Cleavage of talin1 by calpanin2 is responsible for its activation and further mediation of inside-out signaling of integrin β1-containing complex. Furthermore, an increased level of cleaved talin1 is linked with a better response to Yoda1. This effect was the consequence of the calpain2 activation. On the other hand, in HK2 cells with deleted calpain2 cleaved talin1 level remains unchanged. Active cleaved talin1 stimulates integrin clustering on cell membranes [57]. The increase in integrin β1 expression in HK2 cells was caused by Yoda1 treatment and activation of Piezo1 [9]. A similar phenomenon was observed in mice treated with Yoda1. On the other hand, silencing of Piezo1 by siRNA reversed the augmentation of integrin β1 expression within HK2 cells [9]. Deletion of calpain2 in HK2 cells reduced the activation of cells by Piezo1 and decreased integrin β1 capacity [9]. Increased expression of both calpain2 and integrinβ1 was also observed in mouse proximal tubular cells. The former effect was ameliorated by spider peptide toxin (GsMTx4) [9]. Stimulation of the cells by Yoda1 increased the level of phosphorylated (Tyr 397) focal adhesion kinase (p-FAK). P-FAK is the element of downstream signaling of the integrin receptor. In cells with silenced Piezo1 by siRNA treatment, an unchanged concentration of p-FAK after Yoda1 application was observed [9]. All in all, Piezo1 is hypothesized to be the upstream element responsible for the activation of integrin. This pathway is supposed to participate in profibrotic response induction.

Fu et al. [26] proved that increased stiffness of the renal mesangial cell environment aggravates the fibrotic process. In cultures settled on hard substrate, increased ECM deposition was observed. This observation was accompanied by increased translocation of the transcription factor of YAP (Yes-associated Protein) to the nucleus of the cell. The above-mentioned effect was dependent on p38MAPK. Furthermore, in mice with unilateral ureteral obstruction, kidney fibrosis was induced. This effect was reduced by short hairpin RNA (shRNA) targeted to Piezo1 and responsible for lowering the expression of Piezo1 channels. The described method not only reduced fibrosis but also improved kidney function. This study clearly showed that stiff substrate aggravates fibrosis and that this effect is dependent on the Piezo1-YAP-p38MAPK pathway.

## 6. Piezo1 Expression in Renal Fibrosis

The Piezo1 mRNA as well as proteins were detected in rodent nephron and collecting duct of kidney [58]. Moreover, the transcript of Piezo1 was found in proximal convoluted tubule cells [36] and in the proximal tubule of mouse kidney and HK2 cells [59].

Zhao et al. [9] examined during autopsy the expression of Piezo1 channels in kidneys obtained from healthy people (control group) and patients with kidney diseases. Using immunofluorescence, Piezo1 was shown in two investigated groups in kidney tubules, especially in proximal tubular cells (colocalized with AQP1- water channel aquaporin-1) and collecting duct principal cells (colocalized with AQP2). The highest amounts of Piezo1 channels were detected in proximal tubules in fibrotic kidneys. Unilateral ureter obstruction (UUO) or folic acid treatment in mice markedly upregulated the Piezo1 protein in kidneys.

In mice, increased expression of the Piezo1 channels on the third and seventh day after UUO was shown. The Piezo1 capacity was 4-fold greater on day 3 and 7.3-fold greater on day 7 compared to control mice. Thus, pathological processes leading to kidney fibrosis increase Piezo1 protein expression in the kidneys [9]. In the UUO model, the physical stimuli due to retrograde pressure of urine are supposed to be responsible for Piezo1 activation and upregulation.

## 7. GsMTx4 in the Prevention of Renal Fibrosis

Piezo1 inhibition partially prevented renal fibrosis caused by UUO or folic acid treatment [9].

**Figure 4:**
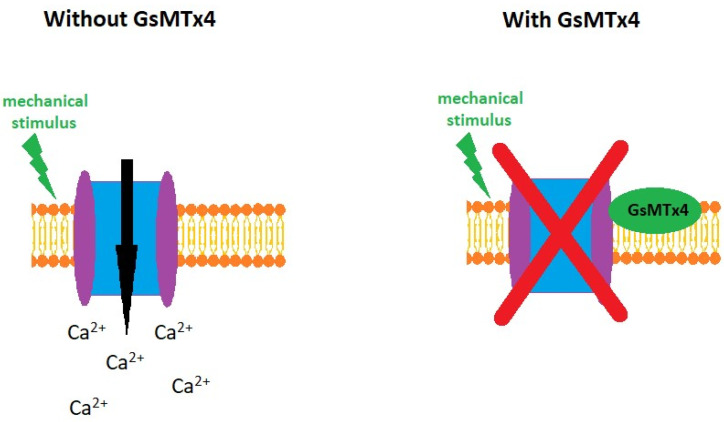
GsMTx4, with its amphipathic structure, attaches itself near the Piezo1 channel. It deactivates the channel, preventing the passage of Ca^2+^ ions in response to mechanical stimuli. As a result, it inhibits the fibrosis pathway depicted in Figure 3 [44].

GsMTx4, the compound isolated from spider venom [35], is an amphipathic peptide toxin that blocks channels sensitive to mechanical stimuli. Furthermore, this peptide has the ability to block Piezo1 ion channels (Figure 4) [44,50]. GsMTx4 modifies the activity of the cation-selective Piezo1 channels erasing the effect of mechanical stimuli [60]. The inhibition of Piezo1 by two enantiomers of GsMTx-4 suggests that their effect is rather more dependent on the modulation of local membrane tension than a direct interaction with the Piezo1 channel [44].

Semi-quantitative immunoblotting showed that the non-specific Piezo1 inhibitor GsMTx4 decreased Piezo1 protein expression on day three after UUO in mice. However, on day seven after UUO, the expression of Piezo1 is unchanged. Masson’s trichrome staining and collagen I immunohistochemistry showed a reduction of fibrosis in mice after UUO on both the third and seventh days. The results report that GsMTx4, at least partially, reduces tubulointerstitial fibrosis in the UUO mice. A similar effect was observed in animals with folic acid-induced nephropathy. GsMTx4 application to the animals reduced the expression of the Piezo1 protein and the fibrotic markers within the kidney were partially decreased. Western blotting confirmed that GsMTx4 lowered the fibronectin and collagen type I content within the kidney [9].

TRPC6 (transient receptor potential cation channel subfamily C member 6) expression was measured as mRNA level was increased after Piezo1 activation by mechanical stimuli. Piezo1 was concluded to respond to mechanical stimuli in concert with other stretch-sensitive channels, including TRPC6 [9]. GsMTx4 has also been shown to block TRPC6 activation by mechanical stimuli [9,61,62].

## 8. The Importance of the Feed-Forward Mechanism in the Case of Piezo1

**Figure 5:**
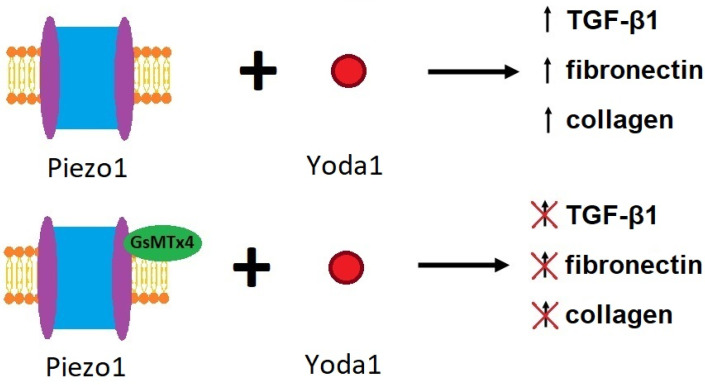
Yoda1 and GsMTx4 effect on Piezo1 and process of fibrosis [9].

During research carried out on mice, inhibition of Piezo1 channels by GsMTx4 and Piezo1 siRNA, as well as Piezo1 gene knockout, resulted in a significant reduction in renal fibrosis. The above data support the statement that Piezo1 is involved in the formation of kidney fibrosis [9,35]. The activation of Piezo1 increases the synthesis and accumulation of extracellular matrix macromolecules resulting in the potentialization of kidney fibrosis. Moreover, in a fibrotic environment, an elevation of Piezo1 molecules was found. A cross-correlation between Piezo1 and TGF-β1 has been noted. TGF-β1, the potent profibrotic cytokine, increases fibrosis in tubulointerstitium. It is able to stimulate the Piezo1 activity and increase 3 fold Piezo channel number, elevating the fibrotic process. This effect was inhibited by GsMTx4. On the other hand, cell exposure to the Piezo1 agonist- Yoda1 increases TGF-β1 concentration (Figure 5). This process strengthens the profibrotic reaction [9].

## 9. A Mechanical Stretch and Fibrosis

In UUO kidneys, increased accumulation of fluid within the tubules elevates tension of the membrane. This process induces profibrotic marker expression in epithelial cells [53]. The cyclic stress of 24 h duration 1.5 fold increases mRNA of Piezo1, as well as 3 fold Piezo1 protein expression. This process is reversed by Piezo1 inhibitors GsMTx4. Moreover, the application of GsMTx4 or siRNA silencing the Piezo1 protein completely suppresses mechanical stress-induced intracellular calcium enhancement. This experiment clearly shows that Piezo1 activity can be induced by mechanical stimuli. Furthermore, mechanical stimuli increase the capacity of α-SMA and fibronectin and decrease epithelial markers such as E-cadherin level. The above changes were not observed after the application of GsMTx4 or siRNA treatment to knock out the Piezo1 [9]. On the other hand, mechanical compression of the HK2 cells also increases Piezo1, α-SMA and fibronectin expression. These fibrotic markers’ elevation is partly inhibited by GsMTx4. The present data suggests that Piezo1 mediates in mechanical stimuli (compression, stretch)-induced fibrosis development [9].

Augmented deposition of extracellular matrix in the interstitial areas causes an increase in tissue stiffness. The experiments performed on cultures of HK2 cells on polyacrylamide gels with different stiffness ranging from 4 kPa to 35 kPa showed the augmentation of Piezo1 expression. This effect was dependent on increasing stiffness of the cellular substrate and it was accompanied by elevation of α-SMA and fibronectin. The response was reversed by GsMTx4. All in all, the Piezo1 channels can detect the stiffness of the extracellular space. Moreover, increased stiffness of the tissue may potentialize fibrosis. This process is dependent on the activity of Piezo1 channels [9].

## 10. Piezo1 Role in Induced Renal Fibrosis Based on Mice Experiment

He Y. et al. [35] studied whether the deletion of Piezo1 gene expression in myeloid cells of mice protects them from developing renal fibrosis. The fibrotic process was evoked in UUO mice treated additionally with folic acid. In Piezo knockout mice expression of fibronectin, vimentin, α-SMA, mRNAs and proteins were significantly lowered compared to wild type [35]. However, the above markers were elevated in wild-type UUO mice treated with folic acid. A mRNA of TGF-β was increased in the model of fibrosis (UUO mice treated with folic acid). This effect is reduced in Piezo1 knockout animals. Furthermore, a similar route was observed for mRNA of Snail1 the transcription factor initiating epithelial–mesenchymal transition the phenomenon driving the fibrosis. The authors [35] concluded that the Piezo1 channel participates in initiating of epithelial–mesenchymal transition that leads to kidney fibrosis. Moreover, Piezo1 increases macrophage infiltration to the fibrotic kidneys. Macrophages participate in the pathogenesis of renal fibrosis [63]. This effect is dependent on chemokines CCL2 and their receptor CCR2. Piezo1 knockout mice showed decreased levels of CCL2 and CCR2 mRNA expression in comparison with their controls, and this effect leads to impaired infiltration of macrophages into the inflammatory area [35]. The Notch signaling pathway also participates in Piezo1-dependent macrophage infiltration. Thus, the Notch receptor intracellular domain, elevated in UUO mice is decreased in Piezo1 knockout mice. Moreover, Piezo1 is involved in regulation of the macrophage activation. Macrophage transformation to M1 and M2 is also dependent on Piezo1. Cytokines comprising tumor necrosis factor-α (TNF-α), interleukin-6 (IL-6), interleukin-1β (IL-1β), nitric oxide synthase 2 (NOS2) typical for activated M1 macrophages and arginase1 (Arg1), interleukin-10 (IL-10) and macrophage mannose receptor1 (MRC1) are associated with activated M2 macrophages [35].

Subsequently, bone marrow-derived macrophages (BMDMs) from Piezo1 knockout mice and those from littermate controls were treated with Yoda1 and lipopolysaccharide to induce inflammatory response. Yoda1 alone was not able to generate expression of inflammatory cytokines (IL-1β, IL-6, TNF-α, NOS2), but, when used combined with lipopolysaccharide, they caused significantly higher elevation of inflammatory cytokines than lipopolysaccharide alone. Decreased activity of calpain in the BMDMs was linked with deficiency of Piezo1. A specific inhibitor of calpain (PD150606) suppressed the expression of inflammatory genes (IL-1β, IL-6, TNF-α, NOS2) in BMDMs subjected to mechanical stretch or Yoda1 combined with lipopolysaccharide [35]. This can lead to the conclusion that Piezo1 controls the inflammatory response of BMDMs through calpain.

## 11. Role of Piezo1 in Development of Vascular Structures

In the process of renal fibrosis, excessive production of collagen and accumulation of ECM leads to restriction of peritubular blood flow and hypoxia of the nephron structures [64]. In kidneys affected by renal fibrosis, we can observe characteristic peritubular capillary rarefaction [65]. Bijkerk et al. showed that induction of vasculogenesis, to sustain proper peritubular capillary density, might protect from ischemic destruction of nephrons within the affected kidneys [66]. Piezo1 channels have a critical role in the development of vascular structure during the embryonic period [21]. Li J et al. observed that global or endothelial-specific disruption of Piezo1 channels was lethal for all mice embryos, while haploinsufficiency led to flaws in mature vessels. Those flaws were recorded as irregular, chaotic alignment of vessels [21]. The presented data showed that Piezo1 channels located in endothelial cells, take part in the development of the vascular system and maintain its proper function as a consequence of receiving frictional forces stimuli generated by fluid flow in vessels [21,67]. Embryos with Piezo1 disruption had significantly lower levels of calpain activity than embryos from the control group. Moreover, specific calpain inhibitors as well as a lack of extracellular Ca^2+^ resulted in impaired alignment of vessels. These data show the critical role of shear-stress-induced Ca^2+^ influx through Piezo1 channels and the activation of calpain in the process of alignment and organization of endothelial cells [21].

## 12. Piezo1 Hypothesis of Renal Anemia

The renal pericytes are erythropoietin-producing cells. Anemia and/or hypoxia are able to induce the expression of Hypoxia Inducible Factor 2α (HIF-2α) [68] that supports erythropoietin synthesis. Furthermore, the in vitro experiments carried out on human renal fibroblasts and medullary interstitial cells showed involvement of HIF-2α in kidney fibrosis development [69]. However, the obtained data are inconsistent because Pan et al. did not confirm HIF-2α involvement in the renal fibrotic process [70].

Chronic kidney disease (CKD) often leads to renal anemia as a result of erythropoietin (EPO) deficiency and other factors such as shortened RBC (red blood cell) life range, iron deficiency and inflammation with higher hepcidin levels [71]. The life of RBC in hemodialyzed patients was observed to be decreased from 120 days to around 50–70 days [71,72]. The uremic milieu was proved to be the cause of shorter RBC life [73]. Increased eryptosis is a result of a rise in intracellular RBC calcium (icCa^2+^). Thus, apoptosis of RBC can be influenced by osmotic stress, reactive oxygen species, and factors that increase icCa^2+^ [74].

Mechanical stimuli activate Piezo1 and Ca^2+^ influx into RBCs. The short-term intracellular (ic) elevation of calcium is important for the erythrocyte shape changes during circulation in capillaries [75]. Mutations in Piezo1 in RBC affect channel inactivation causing longer Ca^2+^ influx and increased icCa^2+^ levels activating the channel for K^+^ ions. This process is responsible for RBC deformation and shrinkage [76]. Yoda1 delays Piezo1 inactivation, which is linked with longer Ca^2+^ influx [46]. Uremic retention solute react with furan fatty acids forming 3-carboxy-4-methyl-5-propyl-2-furanpropanoic acid (CMPF). In addition, the active centers of molecules Jedi1 and Jedi2 are similar to CMPF. These compounds are elevated from 5 to 15 fold compared with healthy people [77].

**Figure 6:**
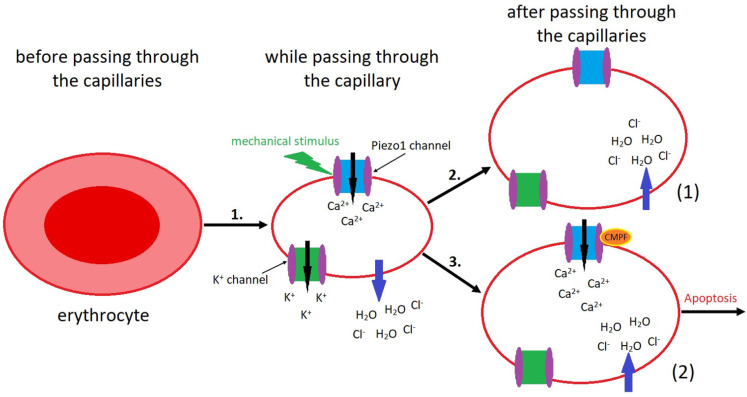
Mechanism of Piezo1-induced erythrocyte deformation and apoptosis in CKD.

Kotanko et al. [40], formed a hypothesis claiming that in patients with chronic kidney disease, CMPF prolongs both activation of Piezo1 and intracellular Ca^2+^ influx (Figure 6). These processes support eryptosis and renal anemia generation. Intervention leading to the lowering of CMPF concentration would be the therapeutic perspective of this anemia treatment. The researchers recommended lowering the concentration of CMPF (the weak lipophilic base) by using different methods. Because of the ineffectiveness of hemodialysis alone, other additional methods are suggested such as the application of adsorptive means following hemodialysis. Albumin-bound CMPF is removed from the plasma by adsorptive material. Then, this plasma is added to the blood cells and subjected to hemodialysis. Moreover, the second method assumes the application of exogenous competitors. This compound is believed to compete with CMPF for albumin binding and results in an increase in free dialyzable CMPF. The third method comprises dialysis of albumins, resulting in the diffusion of free CMPF through the membrane. The blood is dialyzed against the exogenous albumin solution [40]. The described methods would have an important meaning in the development of effective therapy preventing renal anemia.

1. In the process of erythrocyte passage through narrowed blood vessels, mechanical forces exerted by the walls of the constricted vessels act on the erythrocyte. This process results in the activation of Piezo1 channels. Subsequently, it leads to an increased influx of Ca^2+^ ions into the erythrocyte. Elevated calcium ion concentration activates potassium channels, causing K^+^ ions to efflux from the cell. The intracellular environment of the erythrocyte becomes hypotonic relative to the extracellular space, causing water efflux out of the cell correspondingly to the osmotic pressure gradient. This process results in erythrocyte shrinkage and facilitates passage through the narrowed vessel [40].
2. In physiological conditions, after passing the narrowed vessel, an erythrocyte is subjected to lower mechanical forces, leading to the deactivation of Piezo1 channels. Subsequently, intracellular calcium concentration fell and potassium channels closed down. The cellular environment becomes hypertonic, causing water influx into the erythrocyte, as depicted in the first (1) part of Figure 6 [40].
3. In patients with chronic kidney disease (CKD), the augmented concentration of CMPF continuously activates the Piezo1 channels. Consequently, high concentrations of Ca^2+^ ions are accumulated within the erythrocyte, contributing to the initiation of apoptotic processes in this cell. This process ultimately leads to anemia development in individuals with CKD, as illustrated in the second (2) part of Figure 6 [40].

## 13. Summary and Perspectives

The discovery of more specific compounds and regulating the activity of Piezo1 channels is an important purpose of science. Rules of these medicines applications are based on understanding the physiology of the Piezo1 channels, as well as the discovery of their role in the induction of pathological phenomena. The potency of Yoda1 and Jedi 1/2 is limited. However, these compounds play an important role in further research of Piezo1 channel functions. In these investigations, modern methods of gene editing could also be used such as gene silencing (siRNA) or novel methods of genetic engineering allowing genome edition (CRISPR/Cas9). However, it should be remembered that experimentally induced changes of Piezo channel activity are induced not only on the target organ but also in other parts of an organism. These effects can superimpose one onto another. The main aim of further research should focus on the synthesis of effective compounds specific to Piezo channels, allowing for the reduction of their side effects. Due to Piezo2 channels’ localization in certain types of sensory neurons, drugs targeting Piezo2 channels may have applications in mechanical allodynia without affecting the functioning of other systems [50]. On the other hand, the Piezo1 channel is an important regulator of collagen deposition within the kidney and it participates in the development of kidney fibrosis. The Piezo1 channel could be a potential target of pharmacotherapy in kidney diseases.

## Figures and Tables

**Table 1:** Main factors causing renal fibrosis.

Profibrotic Factor	Main Effect	Cell Type/Species	References
TGF-β1	Stimulation of Piezo1 activity and ECM remodeling through increased synthesis of proteins responsible for fibrosis and inhibition of their degradation.	HK2 cells/human, mPTCs/mice	[9]
IFN-γ	Activation of macrophages involved in the process of fibrosis.	Mice	[4]
Folic acid	It disrupts the antioxidant system, which in turn leads to kidney fibrosis.	Mice	[10]
Yoda1	Mimics mechanical stimulation of Piezo1 channels as a selective activator.	Cells with Piezo1 expression	[11]
Ca^2+^	Increase in the concentration of intracellular calcium activates calpain2.	HK2 cells/human	[9]
Calpain2	Activation of talin1.	HK2 cells/human	[9]
Talin1	Increase in the concentration of β1 integrin → fibrosis.	HK2 cells/human	[9,12]
CCL2 and CCR2	Chemokines involved in macrophage chemotaxis.	Inflamed kidneys/mice	[13]

## Data Availability

The data used in this article are sourced from materials mentioned in the References section.

## References

[1] Huang R., Fu P., Ma L. (2023). Kidney fibrosis: From mechanisms to therapeutic medicines. Signal Transduct. Target. Ther..

[2] Schaefer L. (2018). Decoding fibrosis: Mechanisms and translational aspects. Matrix Biol..

[3] Fogo A.B. (2007). Mechanisms of progression of chronic kidney disease. Pediatr. Nephrol..

[4] Lucas T., Waisman A., Ranjan R., Roes J., Krieg T., Müller W., Roers A., Eming S.A. (2010). Differential roles of macrophages in diverse phases of skin repair. J. Immunol..

[5] Duffield J.S. (2014). Cellular and molecular mechanisms in kidney fibrosis. J. Clin. Investig..

[6] Kipari T., Hughes J. (2002). Macrophage-mediated renal cell death. Kidney Int..

[7] Duffield J.S., Lupher M., Thannickal V.J., Wynn T.A. (2013). Host responses in tissue repair and fibrosis. Annu. Rev. Pathol..

[8] Grgic I., Campanholle G., Bijol V., Wang C., Sabbisetti V.S., Ichimura T., Humphreys B.D., Bonventre J.V. (2012). Targeted proximal tubule injury triggers interstitial fibrosis and glomerulosclerosis. Kidney Int..

[9] Zhao X., Kong Y., Liang B., Xu J., Lin Y., Zhou N., Li J., Jiang B., Cheng J., Li C. (2022). Mechanosensitive Piezo1 channels mediate renal fibrosis. JCI Insight..

[10] Li X., Zou Y., Xing J., Fu Y.Y., Wang K.Y., Wan P.Z., Zhai X.Y. (2020). Pretreatment with Roxadustat (FG-4592) Attenuates Folic Acid-Induced Kidney Injury through Antiferroptosis via Akt/GSK-3β/Nrf2 Pathway. Oxid. Med. Cell Longev..

[11] Chubinskiy-Nadezhdin V.I., Vasileva V.Y., Vassilieva I.O., Sudarikova A.V., Morachevskaya E.A., Negulyaev Y.A. (2019). Agonist-induced Piezo1 activation suppresses migration of transformed fibroblasts. Biochem. Biophys. Res. Commun..

[12] Chen C., Manso A.M., Ross R.S. (2019). Talin and Kindlin as Integrin-Activating Proteins: Focus on the Heart. Pediatr. Cardiol..

[13] Tang P.M., Nikolic-Paterson D.J., Lan H.Y. (2019). Macrophages: Versatile players in renal inflammation and fibrosis. Nat. Rev. Nephrol..

[14] Kawasaki H., Ohama T., Hori M., Sato K. (2013). Establishment of Mouse intestinal myofibroblast cell lines. World J. Gastroenterol..

[15] Ricard-Blum S., Baffet G., Théret N. (2018). Molecular and tissue alterations of collagens in fibrosis. Matrix Biol..

[16] Cockerill M., Rigozzi M.K., Terentjev E.M. (2015). Mechanosensitivity of the 2nd Kind: TGF-β Mechanism of Cell Sensing the Substrate Stiffness. PLoS ONE.

[17] Janmey P.A., Miller R.T. (2011). Mechanisms of mechanical signaling in development and disease. J. Cell Sci..

[18] Patel A., Honoré E. (2010). Polycystins and renovascular mechanosensory transduction. Nat. Rev. Nephrol..

[19] Sommerer C., Scharf M., Seitz C., Millonig G., Seitz H.K., Zeier M., Mueller S. (2013). Assessment of renal allograft fibrosis by transient elastography. Transpl. Int..

[20] Arda K., Ciledag N., Aktas E., Aribas B.K., Köse K. (2011). Quantitative assessment of normal soft-tissue elasticity using shear-wave ultrasound elastography. AJR Am. J. Roentgenol..

[21] Li J., Hou B., Tumova S., Muraki K., Bruns A., Ludlow M.J., Sedo A., Hyman A.J., McKeown L., Young R.S. (2014). Piezo1 integration of vascular architecture with physiological force. Nature.

[22] Arishe O.O., Ebeigbe A.B., Webb R.C. (2020). Mechanotransduction and Uterine Blood Flow in Preeclampsia: The Role of Mechanosensing Piezo 1 Ion Channels. Am. J. Hypertens..

[23] Beech D.J., Xiao B. (2018). Piezo channel mechanisms in health and disease. J. Physiol..

[24] Zhao Q., Zhou H., Li X., Xiao B. (2019). The mechanosensitive Piezo1 channel: A three-bladed propeller-like structure and a lever-like mechanogating mechanism. FEBS J..

[25] Volkers L., Mechioukhi Y., Coste B. (2015). Piezo channels: From structure to function. Pflügers Arch.-Eur. J. Physiol.

[26] Fu Y., Wan P., Zhang J., Li X., Xing J., Zou Y., Wang K., Peng H., Zhu Q., Cao L. (2021). Targeting Mechanosensitive Piezo1 Alleviated Renal Fibrosis through p38MAPK-YAP Pathway. Front. Cell Dev. Biol..

[27] Syeda R., Florendo M.N., Cox C.D., Kefauver J.M., Santos J.S., Martinac B., Patapoutian A. (2016). Piezo1 Channels Are Inherently Mechanosensitive. Cell Rep..

[28] Teng J., Loukin S., Anishkin A., Kung C. (2015). The force-from-lipid (FFL) principle of mechanosensitivity, at large and in elements. Pflügers Arch.-Eur. J. Physiol..

[29] Cox C.D., Bae C., Ziegler L., Hartley S., Nikolova-Krstevski V., Rohde P.R., Ng C.A., Sachs F., Gottlieb P.A., Martinac B. (2016). Removal of the mechanoprotective influence of the cytoskeleton reveals PIEZO1 is gated by bilayer tension. Nat. Commun..

[30] Wang J., Jiang J., Yang X., Zhou G., Wang L., Xiao B. (2022). Tethering Piezo channels to the actin cytoskeleton for mechanogating via the cadherin-β-catenin mechanotransduction complex. Cell Rep..

[31] Ellefsen K.L., Holt J.R., Chang A.C., Nourse J.L., Arulmoli J., Mekhdjian A.H., Abuwarda H., Tombola F., Flanagan L.A., Dunn A.R. (2019). Myosin-II mediated traction forces evoke localized Piezo1-dependent Ca^2+^ flickers. Commun. Biol..

[32] Wang S., Chennupati R., Kaur H., Iring A., Wettschureck N., Offermanns S. (2016). Endothelial cation channel PIEZO1 controls blood pressure by mediating flow-induced ATP release. J. Clin. Investig..

[33] Martins J.R., Penton D., Peyronnet R., Arhatte M., Moro C., Picard N., Kurt B., Patel A., Honoré E., Demolombe S. (2016). Piezo1-dependent regulation of urinary osmolarity. Pflügers Arch.-Eur. J. Physiol..

[34] Sun W., Chi S., Li Y., Ling S., Tan Y., Xu Y., Jiang F., Li J., Liu C., Zhong G. (2019). The mechanosensitive piezo1 channel is required for bone formation. eLife.

[35] He Y., Deng B., Liu S., Luo S., Ning Y., Pan X., Wan R., Chen Y., Zhang Z., Jiang J. (2022). Myeloid Piezo1 Deletion Protects Renal Fibrosis by Restraining Macrophage Infiltration and Activation. Hypertension.

[36] Peyronnet R., Martins J.R., Duprat F., Demolombe S., Arhatte M., Jodar M., Tauc M., Duranton C., Paulais M., Teulon J. (2013). Piezo1-dependent stretch-activated channels are inhibited by Polycystin-2 in renal tubular epithelial cells. EMBO Rep..

[37] Li X., Hu J., Zhao X., Li J., Chen Y. (2022). Piezo channels in the urinary system. Exp. Mol. Med..

[38] Mochida Y., Ochiai K., Nagase T., Nonomura K., Akimoto Y., Fukuhara H., Sakai T., Matsumura G., Yamaguchi Y., Nagase M. (2022). Piezo2 expression and its alteration by mechanical forces in mouse mesangial cells and renin-producing cells. Sci. Rep..

[39] Szczot M., Nickolls A.R., Lam R.M., Chesler A.T. (2021). The Form and Function of PIEZO2. Annu. Rev. Biochem..

[40] Kotanko P., Jörg D.J., Grobe N., Zaba C. (2022). The Piezo1 hypothesis of renal anemia. FASEB Bioadv..

[41] Yu J.L., Liao H.Y. (2021). Piezo-type mechanosensitive ion channel component 1 (Piezo1) in human cancer. Biomed. Pharmacother..

[42] Coste B., Xiao B., Santos J.S., Syeda R., Grandl J., Spencer K.S., Kim S.E., Schmidt M., Mathur J., Dubin A.E. (2012). Piezo proteins are pore-forming subunits of mechanically activated channels. Nature.

[43] Zhao Q., Wu K., Geng J., Chi S., Wang Y., Zhi P., Zhang M., Xiao B. (2016). Ion Permeation and Mechanotransduction Mechanisms of Mechanosensitive Piezo Channels. Neuron.

[44] Gnanasambandam R., Ghatak C., Yasmann A., Nishizawa K., Sachs F., Ladokhin A.S., Sukharev S.I., Suchyna T.M. (2017). GsMTx4: Mechanism of Inhibiting Mechanosensitive Ion Channels. Biophys. J..

[45] Maneshi M.M., Ziegler L., Sachs F., Hua S.Z., Gottlieb P.A. (2018). Enantiomeric Aβ peptides inhibit the fluid shear stress response of PIEZO1. Sci. Rep..

[46] Wang Y., Chi S., Guo H., Li G., Wang L., Zhao Q., Rao Y., Zu L., He W., Xiao B. (2018). A lever-like transduction pathway for long-distance chemical- and mechano-gating of the mechanosensitive Piezo1 channel. Nat. Commun..

[47] Syeda R., Xu J., Dubin A.E., Coste B., Mathur J., Huynh T., Matzen J., Lao J., Tully D.C., Engels I.H. (2015). Chemical activation of the mechanotransduction channel Piezo1. eLife.

[48] Deivasikamani V., Dhayalan S., Abudushalamu Y., Mughal R., Visnagri A., Cuthbertson K., Scragg J.L., Munsey T.S., Viswambharan H., Muraki K. (2019). Piezo1 channel activation mimics high glucose as a stimulator of insulin release. Sci. Rep..

[49] Evans E.L., Cuthbertson K., Endesh N., Rode B., Blythe N.M., Hyman A.J., Hall S.J., Gaunt H.J., Ludlow M.J., Foster R. (2018). Yoda1 analogue (Dooku1)which antagonizes Yoda1-evoked activation of Piezo1 and aortic relaxation. Br. J. Pharmacol..

[50] Xiao B. (2020). Levering Mechanically Activated Piezo Channels for Potential Pharmacological Intervention. Annu. Rev. Pharmacol. Toxicol..

[51] Wijerathne T.D., Ozkan A.D., Lacroix J.J. (2022). Yoda1’s energetic footprint on Piezo1 channels and its modulation by voltage and temperature. Proc. Natl. Acad. Sci. USA.

[52] Gu Y.Y., Liu X.S., Huang X.R., Yu X.Q., Lan H.Y. (2020). Diverse Role of TGF-β in Kidney Disease. Front. Cell Dev. Biol..

[53] Tan R.J., Zhou D., Liu Y. (2016). Signaling Crosstalk between Tubular Epithelial Cells and Interstitial Fibroblasts after Kidney Injury. Kidney Dis..

[54] Yuan Q., Ren Q., Li L., Tan H., Lu M., Tian Y., Huang L., Zhao B., Fu H., Hou F.F. (2022). A Klotho-derived peptide protects against kidney fibrosis by targeting TGF-β signaling. Nat. Commun..

[55] Zhang Y., Su S.A., Li W., Ma Y., Shen J., Wang Y., Shen Y., Chen J., Ji Y., Xie Y. (2021). Piezo1-mediated mechanotransduction promotes cardiac hypertrophy by impairing calcium homeostasis to activate calpain/calcineurin signaling. Hypertension.

[56] Liu S., Xu X., Fang Z., Ning Y., Deng B., Pan X., He Y., Yang Z., Huang K., Li J. (2021). Piezo1 impairs hepatocellular tumor growth via deregulation of the MAPK-mediated YAP signaling pathway. Cell Calcium.

[57] Klapholz B., Brown N.H. (2017). Talin—The master of integrin adhesions. J. Cell Sci..

[58] Dalghi M.G., Clayton D.R., Ruiz W.G., Al-Bataineh M.M., Satlin L.M., Kleyman T.R., Ricke W.A., Carattino M.D., Apodaca G. (2019). Expression and distribution of PIEZO1 in the mouse urinary tract. Am. J. Physiol. Renal Physiol..

[59] Khundmiri S.J., Chen L., Lederer E.D., Yang C.R., Knepper M.A. (2021). Transcriptomes of Major Proximal Tubule Cell Culture Models. J. Am. Soc. Nephrol..

[60] Bae C., Sachs F., Gottlieb P.A. (2011). The mechanosensitive ion channel Piezo1 is inhibited by the peptide GsMTx4. Biochemistry.

[61] Dryer S.E., Roshanravan H., Kim E.Y. (2019). TRPC channels: Regulation, dysregulation and contributions to chronic kidney disease. Biochim. Biophys. Acta Mol. Basis Dis..

[62] MAnderson E.Y., Kim H., Hagmann T., Benzing S.E. (2013). Dryer Opposing effects of podocin on the gating of podocyte TRPC6 channels evoked by membrane stretch or diacylglycerol. Am. J. Physiol. Cell Physiol..

[63] Jiang Y., Wang Y., Ma P., An D., Zhao J., Liang S., Ye Y., Lu Y., Zhang P., Liu X. (2019). Myeloid-specific targeting of Notch ameliorates murine renal fibrosis via reduced infiltration and activation of bone marrow-derived macrophage. Protein Cell.

[64] Humphreys B.D. (2018). Mechanisms of Renal Fibrosis. Annu. Rev. Physiol..

[65] Bohle A., Strutz F., Müller G.A. (1994). On the pathogenesis of chronic renal failure in primary glomerulopathies: A view from the interstitium. Exp. Nephrol..

[66] Bijkerk R., van Solingen C., de Boer H.C., van der Pol P., Khairoun M., de Bruin R.G., van Oeveren-Rietdijk A.M., Lievers E., Schlagwein N., van Gijlswijk D.J. (2014). Hematopoietic microRNA-126 protects against renal ischemia/reperfusion injury by promoting vascular integrity. J. Am. Soc. Nephrol..

[67] Chiu J.J., Chien S. (2011). Effects of disturbed flow on vascular endothelium: Pathophysiological basis and clinical perspectives. Physiol. Rev..

[68] Chang Y.T., Yang C.C., Pan S.Y., Chou Y.H., Chang F.C., Lai C.F., Tsai M.H., Hsu H.L., Lin C.H., Chiang W.C. (2016). DNA methyltransferase inhibition restores erythropoietin production in fibrotic murine kidneys. J. Clin. Investig..

[69] Norman J.T., Clark I.M., Garcia P.L. (2000). Hypoxia promotes fibrogenesis in human renal fibroblasts. Kidney Int..

[70] Pan S.-Y., Tsai P.-Z., Chou Y.-H., Chang Y.-T., Chang F.-C., Chiu Y.-L., Chiang W.-C., Hsu T., Chen Y.-M., Chu T.-S. (2021). Kidney pericyte hypoxia-inducible factor regulates erythropoiesis but not kidney fibrosis. Kidney Int..

[71] Babitt J.L., Eisenga M.F., Haase V.H., Kshirsagar A.V., Levin A., Locatelli F., Małyszko J., Swinkels D.W., Tarng D.-C., Cheung M. (2021). Controversies in optimal anemia management: Conclusions from a Kidney Disease: Improving Global Outcomes (KDIGO) Conference. Kidney Int..

[72] Vos F.E., Schollum J.B., Coulter C.V., Doyle T.C., Duffull S.B., Walker R.J. (2011). Red blood cell survival in long-term dialysis patients. Am. J. Kidney Dis..

[73] Loge J.P., Lange R.D., Moore C.V. (1958). Characterization of the anemia associated with chronic renal insufficiency. Am. J. Med..

[74] Bissinger R., Bhuyan A.A.M., Qadri S.M., Lang F. (2019). Oxidative stress, eryptosis and anemia: A pivotal mechanistic nexus in systemic diseases. FEBS J..

[75] Cahalan S.M., Lukacs V., Ranade S.S., Chien S., Bandell M., Patapoutian A. (2015). Piezo1 links mechanical forces to red blood cell volume. eLife.

[76] Albuisson J., Murthy S.E., Bandell M., Coste B., Louis-Dit-Picard H., Mathur J., Fénéant-Thibault M., Tertian G., de Jaureguiberry J.P., Syfuss P.Y. (2013). Dehydrated hereditary stomatocytosis linked to gain-of-function mutations in mechanically activated PIEZO1 ion channels. Nat. Commun..

[77] Vanholder R., De Smet R., Glorieux G., Argilés A., Baurmeister U., Brunet P., Clark W., Cohen G., De Deyn P.P., Deppisch R. (2003). Review on uremic toxins: Classification, concentration, and interindividual variability. Kidney Int..

